# Geriatric Nutritional Risk Index (GNRI) Independently Predicts Amputation Inchronic Criticallimb Ischemia (CLI)

**DOI:** 10.1371/journal.pone.0152111

**Published:** 2016-03-24

**Authors:** Han Luo, Hongliu Yang, Bin Huang, Ding Yuan, Jingqiang Zhu, Jichun Zhao

**Affiliations:** 1 Department of Nephrology, Biostatistics Center, West China Hospital, Sichuan University, Chengdu, China; 2 Department of Vascular Surgery, West China Hospital, Sichuan University, Chengdu, China; 3 Department of Thyroid & Parathyroid Surgery, West China Hospital, Sichuan University, Chengdu, China; Harefield Hospital, UNITED KINGDOM

## Abstract

**Objective:**

General malnutrition usually occurs in critical limb ischemia (CLI) patients because of shortness of appetite and sleeplessness leaded by chronic pain. And amputation frequently is end-point of CLI patients. So the aim of this study was to assess the predictive ability of Geriatric nutritional risk index (GNRI) for predicting amputation in patients with CLI.

**Methods:**

A retrospective study was designed. Demographics, history, comorbidity, and risk factors for peripheral vascular disease of admitted patients, and laboratory study were documented. Patients’ height, weight and BMI were recorded. Amputation was identified as end-point during follow-up. Patients’ amputation-free survival (AFS) was recorded.

**Result:**

172 patients were identified, with mean age 71.98±3.12. Geriatric nutritional risk index (GNRI) = 90 was taken as cutoff value of high risk of amputation for CLI patients via using receiver operating characteristic (ROC) curve. Span of follow-up was 12–48 months. During follow-up, 60 patients (36.04%) received amputation surgery. And analyzed by Cox proportional hazards model, it is found that GNRI was the independent predictive factor for amputation in long term.

**Conclusion:**

This study revealed that GNRI was a reliable and effective predictive marker for AFS. GNRI could identify patients with high risk for amputation in early time.

## Introduction

Critical limb ischemia (CLI) is a common disease in old patients, and though there is no scientific data about incidence rate in Chinese people, yet it is up to 0.35% in American, according to retrospective analysis.[1]Thus it is estimated that more than 4.5 million people would be influenced by CLI in China. Because of chronic resting pain, patients usually appear to be of anorexia and sleepless, which leads to general malnutrition[2,3] (it is a common comorbidity in CLI patients). And it is recorded that amputation occurred in more than quarter of patients in one year if no treatment. Moreover, risk factor analysis has revealed that malnutrition is a risk factor [4,5]following smoking, drinking, and diabetic mellitus for CLI. So most of CLI patients suffer from emaciation and amputation, but whether the two indexes have a determined causal link is uncertain.

In recent years, Geriatric nutritional risk index (GNRI) is universally adopted to evaluate patients’ nutrition condition, which is an effective and simple risk index to present patients’ nutritional risk and has been proved to be a determined predictive index for prognosis in aged, dialysis, cardiovascular patients and healthcare[6–9]. However, GNRI is nearly never adopted to evaluate condition of patients suffering from vascular disease. Moreover, risk stratification in early time becomes more and more important for patients with CLI, because it is useful for clinical decision making and offering in-time and impacted therapy, then improve patients’ life quality.

Therefore, a retrospective study would be conducted to adopt GNRI to evaluate CLI patients’ nutrition condition and predict prognosis.

### Patients and methods

Patients included in this study were recruited from Vascular Department of West China Hospital, Sichuan University between Mar.2010 and Jan.2013.

According to the Trans-Atlantic Inter-Society Consensus Document on Management of Peripheral Arterial Disease (TASC) criteria[10], objective criteria[4]for admitted patients that support the diagnosis of CLI includes an ankle-brachial index of 0.4 or less, an ankle systolic pressure of 50 mm Hg or less, or a toe systolic pressure of 30 mm Hg or less.Moreover, included patients’ ischemic symptoms presented for more than 4 weeks.

Exclusion criteria: a. age<65 years; b. patients with determined evidence of acute limb ischemia and necrosis; c. hyponatremia (<135 mmol/L) and hypernatremia (>145 mmol/L), d. severe hepatic disease and severe renal insufficiency (creatinine clearance calculated from the Cockroft and Gault formula at <15 mL/min[11]) to rule out non-malnutrition-related modifications in albumin.

### GNRI identification

When admission, indexes including demographics (age, gender), physical parameter [eight, weight, body mass index (BMI)], and history (smoking, drug, alcohol abuse), and comorbidity and laboratory studies (albumin, lymphocyte etc) were documented in standardized database by nurses and doctors. Each patient’s treatment process including procedure (like bypass, intervention, angioplasty and amputation) and drugs was recorded. All patients’ nutrition condition was evaluated according to GNRI formula[12]: GNRI = [1.489*albumin (g/L)] +[41.7 *(weight/WLo)], where WLo means ideal weight and was calculated from the Lorentz equations: for men: H—100—[(H—150)/4]; for women: H—100—[(H—150)/2.5](H: height). From these GNRI values, 4 grades of nutrition-related risk was graded according to previous research[12]: high risk (GNRI: <82), moderate risk (GNRI: 82 to <92), low risk (GNRI: 92 to ≤98), and no risk (GNRI: >98).

Discharged patients were followed up by outpatient consultation or call. Each patient had same standard follow-up form and was followed at end of 1st, 3rd, 6th, and 12th months after discharged, then once time per year thereafter. Call follow-up was at the same interval. In follow-up form, ischemic symptom and sign (pain, claudication, necrosis et al) and whether amputated (time and level) was focus. In this study, primary end-point was identified as amputation (major or minor) or 36 months later after discharged.

### Ethics

The study does not need the approvement of the Ethics Committee of West China Hospital, Sichuan University.

### Analysis method

The effect of GNRI on outcome was analyzed by receiver operating characteristic (ROC) curve with amputation as the primary variable. The chi-square test was used to perform univariate analysis for categorical variables and student’s T test for continuous variables. Survival analysis was conducted through Kaplan-Meier survival curves, and differences were compared using the log-rank test. Cox proportional hazards model was conducted in a stepwise fashion. Analysis was performed using SPSS version 16 (SPSS Inc, Chicago, IL). P < .05 was considered significantly different.

## Result

215 patients met the inclusion criteria for this study. Four patients lost because of death before end-point of follow-up and 43 patients who met exclusion criteria were excluded from final analysis. At last, 172 patients were included into final outcome analysis. Span of follow-up was 12–48 months (mean duration: 35.8 months).

### Cutoff value of GNRI selected

**Fig 1:**
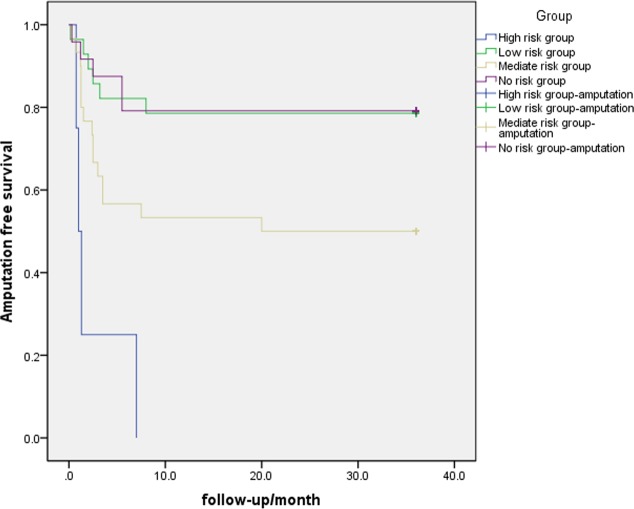
The graph shows the amputation-free survival (AFS) in High risk (H)/Low risk (L)/Moderate risk (M)/No risk (No) group respectively. The difference between groups was significant (log-rank test, P < .05).

After admission, GNRI value was calculated for each included patients. According to GNRI gradation, inclusion patients were divided into 4 groups: high/moderate/low/no risk group (8/60/56/48 respectively)[12]. Number of patients received amputation in 4 groups was 8 (100%), 30(50%), 12 (21.4%) and 10 (20.8%) in high/moderate/low/no risk group respectively. Thus incidence of amputation increases significantly with risk gradation increasing. In another word, AFS in 4 groups was 0, 50%, 78.6%, and 79.2% successively. Overall survival analysis has significant difference between 4 groups (p = 0.001). Survival analysis for the 4 groups was shown in Fig 1.

**Fig 2:**
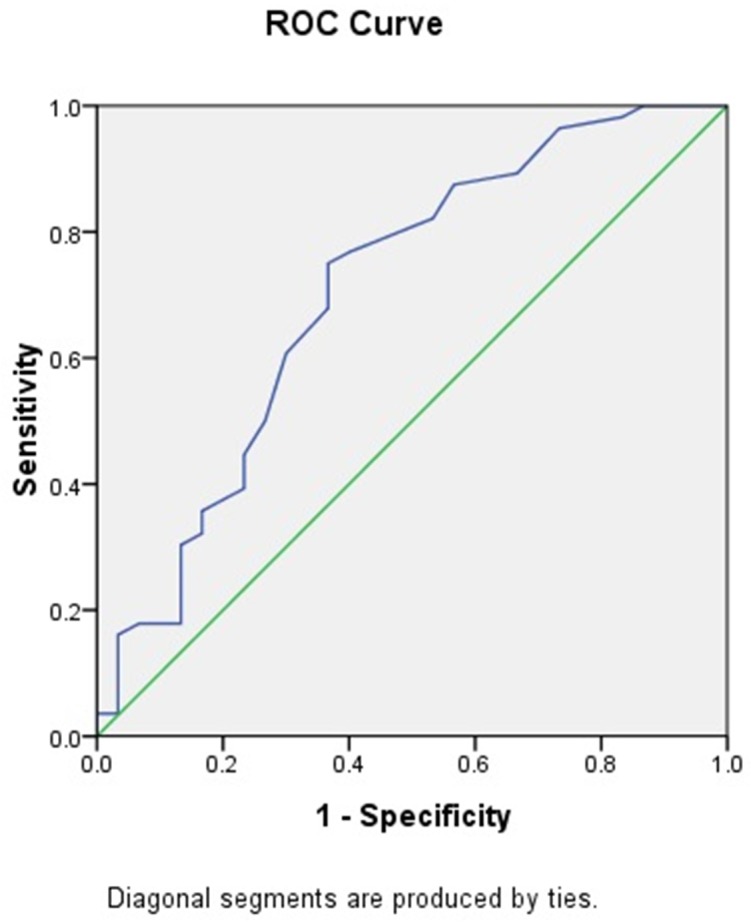
c-statistic (area under curve) = .705.

However, GNRI appeared to have an obvious influence on outcome in continuous fashion. So a cutoff value of GNRI needs to be detracted to better guide clinical practice. ROC curve was used to identify cutoff value and shown in Fig 2. In ROC analysis setting, AFS was set as state variable. c-statistic (area under curve) was 0.705. GNRI = 90 was selected as cutoff value with maximum discriminative power (sensitivity 75%, specificity 63%). 106 patients (61.6%) had GNRI≥90, 66 patients had GNRI<90.

### Independent risk factor identification

**Fig 3:**
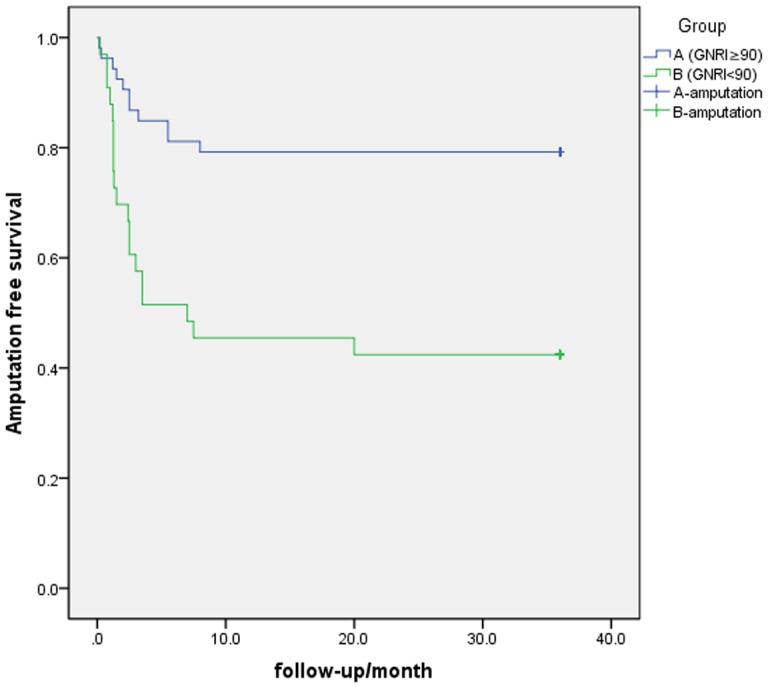
The graph shows the amputation-free survival (AFS) in group A (GNRI≥90), and group B (GNRI<90), The difference between group A and group B was significant (log-rank test, P < .05).

According cutoff value (GNRI = 90), patients were divided into two groups: group A (GNRI≥90) group B (GNRI<90). Survival comparison between group A and B was shown in Fig 3. Older patients appeared to have a worse nutrition condition significantly (P = .016) with mean age in group A versus group B: 69.92±2.68 vs 75.27±3.29. Albumin (ALB, 38.72±3.22 vs 35.36±4.75), BMI (22.79±3.19 vs 20.05±2.28), low density lipoprotein (LDL, 2.57±0.69 vs 2.24±0.85) and cholesterol (CHOL, 4.33±0.83 vs3.93±1.18) and diabetic mellitus(DM) had a significant difference (P = .001/.000/.006/.011/.024 respectively) between group A versus group B. Mortality in both groups was 2 (P = .638). Other laboratory test, comorbidity percentage, social status, living condition and Fontaine gradation had no significant difference. Baseline comparison was shown Table 1. Procedure comparison of two groups was shown in Table 2. Amputation rate in group A (GNRI≥90) and B (GNRI<90) was 20.8% and 57.6% respectively and significant difference was found (P < .001). Detail of amputated patients was listed in Table 3.

**Table 1:** Baseline comparison between group A and B.

Index	Group A	Group B	P value^d^
Age	69.92±10.68	75.27±8.29	.016
BMI ^a^	22.79±3.19	20.05±2.28	< .001
ALB	38.72±3.22	35.36±4.75	.001
LDL	2.57±0.69	2.24±0.85	.006
CHOL	4.33±0.83	3.93±1.18	.011
WBC	8.51±3.75	9.44±4.03	NS
K	3.89±0.45	3.90±0.56	NS
CRE	85.06±27.40	82.15±33.51	NS
Neutrophil	6.27±3.34	6.45±2.57	NS
Lymphocyte	1.57±0.63	1.38±0.61	NS
HDL	1.15±0.32	1.13±0.47	NS
TG	1.56±1.13	1.68±1.26	NS
Gender (male/female)	78/28	44/22	NS
Smoking	62	38	NS
Hypertension ^b^	54	42	NS
DM	24	6	NS
Surgery	56	26	NS
Heart disease	30	14	NS
CAD	14	10	NS
Bone disease	2	2	NS
Social status^c^			NS
Bachelor and above	12	8	
Middle school	76	43	
Primary school	18	15	
Living condition			NS
Nursing home	21	14	
Private home	85	52	
Fontaine gradation			NS
II	4	2	
III	70	44	
IV	32	20	

a. Body mass index (BMI), Albumin (ALB), BMI, low density lipoprotein (LDL), cholesterol (CHOL), white blood cell (WBC),potassium (K), creatinine (CRE), high density lipoprotein (HDL), triglyceride (TG), coronary artery disease (CAD), diabetic mellitus (DM).

b. Comorbidity was identified by history mainly.

c. Social status was presented by educational level

d. P < .05 was considered significant different, NS: no significance.

**Table 2:** Procedure comparison in group A and B.

Procedure	Group A (GNRI≥90)	Group B (GNRI<90)	P value ^b^
Surgery	34	14	NS
Angioplasty/stent	20	11	NS
Hybrid ^a^	2	1	NS
Conservative	50	40	NS

a. Hybrid: surgery combines with angioplasty/stenting

b. P<0.05 was considered significant different, NS: no significance.

**Table 3:** Amputation comparison between group A and B.

	Group A (GNRI≥90)	Group B (GNRI<90)	P value ^b^
Amputation	22	38	< .001
AKA ^a^	1	1	NS
BKA	9	13	.037
Toe	12	24	< .001

a. AKA: above-knee amputation; BKA: below-knee amputation.

b. P<0.05 was considered significant different, NS: no significance.

Risk factors for CLI patients: age, albumin, BMI, DM and GNRI, LDL, CHOL were included into multivariate analysis (Cox proportional hazards model). Age, GNRI and albumin were independent predictive factors for amputation (hazard ratios [95% confidence interval], 1.050 [1.018,1.083],P = .002;0.953 [0.916, 0.991], P = .016; 0.923 [0.862, 0.988], P = .021 respectively). It is shown in Table 4.

**Table 4:** Multivariate analysis of factors affecting overall amputation in patients with critical limb ischemia.

Index	Hazard ratio	95% confidence interval	P value ^b^
Age	1.050	1.018,1.083	.002
GNRI ^a^	0.953	0.916, 0.991	.016
ALB	0.923	0.862, 0.988	.021
BMI/DM/LDL/CHOL			NS

a. Geriatric nutritional risk index (GNRI), albumin (ALB), body mass index (BMI), low density lipoprotein (LDL), cholesterol (CHOL), diabetes mellitus (DM).

b. P < .05 was considered significant different, NS: no significance.

## Discussion

This study shows that GNRI is an effective and simple risk stratification marker for patients presenting with CLI. Decreased GNRI (<90) represents a high risk for amputation.

It is said that major lower limbs amputation [above-the-knee amputation (AKA) or below-the-knee amputation (BKA)] at 1 year occur in approximate 20% of untreated CLI patients.[13–15] In this study, overall amputation rate is 34.9%, and in group A (GNRI≥90) and B (GNRI<90) was 20.8% and 57.6% respectively. Moreover, it is revealed in Table 3 that CLI patients with wore nutritional condition (in Group B) intend to have minor amputation (BKA and toe amputation). It is an obvious tendency that incidence of amputation would be higher (a worse prognosis) in patients with a worse nutrition condition. This tendency is in accordance with outcome in other fields, like nephrology, postoperative management and oncology etc. [6,9,16,17]

Aged patients easily suffer from malnutrition[18], especially when aged patients present with CLI, because of ischemic rest pain and sleeplessness, which often is classically described as a burning pain in the joint of the foot and toes that is worse at night when the patient is in bed, incidence rate of malnutrition is relatively high[2,3]. In this study, patients in group B (GNRI<90) are older than in group A (GNRI≥90), naturally older patients have a worse nutrition condition. Therefore, variable of age could predict prognosis in CLI patients via affecting nutrition condition. And albumin is a crucial index for nutrition condition and important factor for GNRI formula conduction. So variable of albumin also affects nutrition condition to predict prognosis in this study. In all, essence of three independent risk factors is nutrition condition, which is evaluated by GNRI.

Moreover, GNRI is a simple and effective marker to identify patients with nutrition risk, which has been approved to be an independent predictive index for prognosis in dialysis patients.[6,16] Though GNRI is nearly firstly used to predict prognosis in CLI patients, and cutoff value is found, yet unfortunately there is no randomized controlled trial (RCT) to confirm this result. So a RCT: evaluating CLI patients’ long-term prognosis with or without nutrition support will be designed in our institution to confirm this outcome.

In previous researches [19–22], revascularization procedure (surgery and angioplasty/stenting) could have a determined better prognosis than conservative therapy. However, procedure percentage (48.8%) is relative low for CLI patients in our institution, especially in group B (GNRI<90, 36.4%). CLI patients in our institution usually suffer from extremely severe ischemia and pain when admitted or transferred from local hospitals. After long-term exhaustion, patients usually suffer from severe general malnutrition, which leads to a weak tolerance for procedure. That is why relative high proportion of CLI patients received non-procedure therapy. Nutrition support may enhance patients’ tolerance for procedure, which is also beneficial for patients. Furthermore, in recent research [23], it was approved that underweight patients with CLI have an extremely poor prognosis. And low BMI was identified as an independent predictor of a poor prognosis in patients with CLI. That was similar to the conclusion in the present study.

Risk stratification utilizing simple clinical data to identify patients with high or low risk is meaningful in clinical practice. In this study, GNRI is adopted to evaluate and grade patients, but which is not designed according to vascular disease [12]. If an exact formula is calculated for vascular disease specifically, a more accurate evaluation could be expected to inform clinical practice.

## Conclusion

In conclusion, GNRI is an effective predictive marker for AFSin patients presenting with CLI in long term. Patients with GNRI<90 are easier to suffer from amputation, especially minor amputation compared with GNRI≥90. Moreover, GNRI could identify patients with high risk for amputation in early time, and if patients with GNRI<90 could have in-time nutrition support, AFS may be longer, even avoid amputation, but which needs further research to confirm.
